# 

**Published:** 1897-12-25

**Authors:** 


					THE HOSPITAL. ABSOLUTELY PURE, therefore BEST. Dec. 25th:, 1897.
CADBURY'S HLJF CADBURY'S
oocoa .FTP |rf 1Br? cocoa
'SS^2!^S'P"',y'',P',!",t % ?MSsi? MO ALKALIES USED.
RW1CH HOSPI.TAL
No. 587. Yol. XXIII. [J3KEJS Satueday,
Deo. 25th, 1897.
(-SnbscriptionTorms,] pRICE TWOPENCE.

				

